# The scalable precision medicine open knowledge engine (SPOKE): a massive knowledge graph of biomedical information

**DOI:** 10.1093/bioinformatics/btad080

**Published:** 2023-02-09

**Authors:** John H Morris, Karthik Soman, Rabia E Akbas, Xiaoyuan Zhou, Brett Smith, Elaine C Meng, Conrad C Huang, Gabriel Cerono, Gundolf Schenk, Angela Rizk-Jackson, Adil Harroud, Lauren Sanders, Sylvain V Costes, Krish Bharat, Arjun Chakraborty, Alexander R Pico, Taline Mardirossian, Michael Keiser, Alice Tang, Josef Hardi, Yongmei Shi, Mark Musen, Sharat Israni, Sui Huang, Peter W Rose, Charlotte A Nelson, Sergio E Baranzini

**Affiliations:** Department of Pharmaceutical Chemistry, School of Pharmacy, University of California, San Francisco, San Francisco, CA 94158, USA; Department of Neurology, Weill Institute for Neurosciences, University of California, San Francisco, San Francisco, CA 94158, USA; Department of Neurology, Weill Institute for Neurosciences, University of California, San Francisco, San Francisco, CA 94158, USA; Department of Neurology, Weill Institute for Neurosciences, University of California, San Francisco, San Francisco, CA 94158, USA; Institute for Systems Biology, Seattle, WA 98109, USA; Department of Pharmaceutical Chemistry, School of Pharmacy, University of California, San Francisco, San Francisco, CA 94158, USA; Department of Pharmaceutical Chemistry, School of Pharmacy, University of California, San Francisco, San Francisco, CA 94158, USA; Department of Neurology, Weill Institute for Neurosciences, University of California, San Francisco, San Francisco, CA 94158, USA; Bakar Computational Health Sciences Institute, University of California, San Francisco, San Francisco, CA 94158, USA; Bakar Computational Health Sciences Institute, University of California, San Francisco, San Francisco, CA 94158, USA; Department of Neurology, Weill Institute for Neurosciences, University of California, San Francisco, San Francisco, CA 94158, USA; Space Biosciences Division, NASA Ames Research Center, Moffett Field, CA 94035, USA; Space Biosciences Division, NASA Ames Research Center, Moffett Field, CA 94035, USA; Department of Neurology, Weill Institute for Neurosciences, University of California, San Francisco, San Francisco, CA 94158, USA; Department of Neurology, Weill Institute for Neurosciences, University of California, San Francisco, San Francisco, CA 94158, USA; Data Science and Biotechnology, Gladstone Institutes, University of California, San Francisco, San Francisco, CA 94158, USA; Department of Pharmaceutical Chemistry, University of California, San Francisco, San Francisco, CA 94143-2550, USA; Department of Pharmaceutical Chemistry, University of California, San Francisco, San Francisco, CA 94143-2550, USA; UCSF-UC Berkeley Bioengineering Graduate Program, University of California, San Francisco, San Francisco, CA 94158, USA; Stanford Center for Biomedical Informatics Research, Stanford University, Stanford, CA 94305-5479, USA; Bakar Computational Health Sciences Institute, University of California, San Francisco, San Francisco, CA 94158, USA; Stanford Center for Biomedical Informatics Research, Stanford University, Stanford, CA 94305-5479, USA; Bakar Computational Health Sciences Institute, University of California, San Francisco, San Francisco, CA 94158, USA; Institute for Systems Biology, Seattle, WA 98109, USA; San Diego Supercomputer Center, University of California, San Diego, La Jolla, CA 92093, USA; Department of Neurology, Weill Institute for Neurosciences, University of California, San Francisco, San Francisco, CA 94158, USA; Department of Neurology, Weill Institute for Neurosciences, University of California, San Francisco, San Francisco, CA 94158, USA

## Abstract

**Motivation:**

Knowledge graphs (KGs) are being adopted in industry, commerce and academia. Biomedical KG presents a challenge due to the complexity, size and heterogeneity of the underlying information.

**Results:**

In this work, we present the Scalable Precision Medicine Open Knowledge Engine (SPOKE), a biomedical KG connecting millions of concepts via semantically meaningful relationships. SPOKE contains 27 million nodes of 21 different types and 53 million edges of 55 types downloaded from 41 databases. The graph is built on the framework of 11 ontologies that maintain its structure, enable mappings and facilitate navigation. SPOKE is built weekly by python scripts which download each resource, check for integrity and completeness, and then create a ‘parent table’ of nodes and edges. Graph queries are translated by a REST API and users can submit searches directly via an API or a graphical user interface. Conclusions/Significance: SPOKE enables the integration of seemingly disparate information to support precision medicine efforts.

**Availability and implementation:**

The SPOKE neighborhood explorer is available at https://spoke.rbvi.ucsf.edu.

**Supplementary information:**

Supplementary data are available at *Bioinformatics* online.

## 1 Introduction

**Fig. 1.**
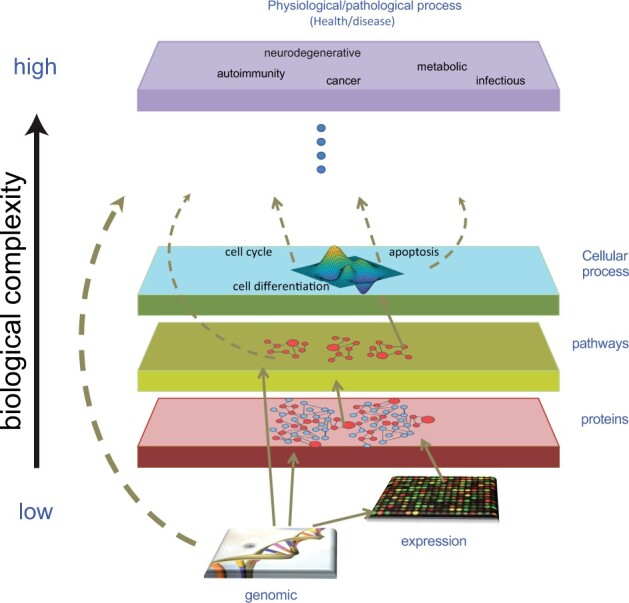
Hierarchical organization of biological complexity. Biomedical information is largely compartmentalized according to disciplines. Integration of information may lead to the emergence of knowledge

Data lead to information, and information leads to knowledge (Ackoff, 1989). Vast amounts of data are being produced at a breathtaking pace (Reinsel *et al.*, 2018), and this explosion in the amount of generated data is causing the number and size of databases and repositories to increase exponentially. In the biomedical domain, this big data problem gets further compounded by the resulting compartmentalization of data resources according to specialty, likely driven by the enormous biological complexity underlying human physiology (Fig. 1).

Even where data and factual knowledge are stored in public repositories, their access and interpretation are still limited by physical, technical and thematic compartmentalization, making it difficult if not impossible for medical professionals to utilize this body of information and connect the dots to facilitate the emergence of knowledge.

Given the complexity of existing relationships among different biomedical fields, graph databases have recently gained popularity as a practical solution to integrate such disparate sources of information. Knowledge graphs with biomedical content have been developed using a variety of strategies, content and target applications (Fecho *et al.*, 2021; Mattingly *et al.*, 2006; Santos *et al.*, 2022).

The scalable precision medicine open knowledge engine (SPOKE) is a knowledge graph that connects information from 41 specialized databases, structured as 21 different node types and 55 edge types, ranging from molecular and cellular biology to pharmacology and clinical practice. SPOKE was conceived with the philosophy that if relevant information is connected, it can result in the emergence of knowledge, and hence provide insights into the understanding of diseases, discovering of drugs and proactively improving personal health.

## 2 Materials and methods

### 2.1 Construction and enrichment of SPOKE

SPOKE currently uses 41 different data sources to construct the knowledge graph (Table 1) although new databases are being added continually. To construct SPOKE, a script downloads and processes each data source on a weekly basis. (See Supplementary material for a detailed description of databases and modeling.)

**Table 1.** SPOKE nodes

Node	Label	Description	Count	Source
1	Anatomy	Tissue (from UBERON)	15 239	http://obophenotype.github.io/uberon/
2	AnatomyCellType	Intermediate node built by combining cell type and anatomy	102	N/A
3	BiologicalProcess	From Gene Ontolology	13 343	http://geneontology.org
4	CellType	From Gene Ontolology	54	https://www.ebi.ac.uk/ols/ontologies/cl
5	CellularComponent	From Gene Ontolology	1722	http://geneontology.org
6	Compound	pharmacological or metabolic compound	2 112 091	https://www.ebi.ac.uk/chembl/
7	Disease	Disease	10 932	https://disease-ontology.org
8	EC	Enzymatic activity	8 287	https://iubmb.qmul.ac.uk/enzyme/
9	Food	Food	992	https://foodon.org
10	Gene	Gene (Entrez)	20 086	https://www.ncbi.nlm.nih.gov/gene
11	MolecularFunction	FROM gene ontolology	3488	http://geneontology.org
12	Nutrient	Nutrient	39	https://www.ebi.ac.uk/chembl/
13	Organism	Organism (NCBI taxonomy)	10 030	https://www.ncbi.nlm.nih.gov/taxonomy
14	Pathway	Biological pathway	3454	https://reactome.org
15	PharmacologicClass	Pharmacological class	577	https://www.ebi.ac.uk/chembl/
16	Protein	Protein (UniProt)	24 805 918	https://www.uniprot.org
17	ProteinDomain	Protein domain (Pfam)	19 178	https://pfam.xfam.org
18	ProteinFamily	Protein family (Pfam)	645	https://pfam.xfam.org
19	Reaction	Metabolic reaction (KEGG or Metacyc)	22 370	https://www.kegg.jp ‖| https://metacyc.org
20	SideEffect	Compound side effect (SIDER)	6061	http://sideeffects.embl.de
21	Symptom	Symptom (MeSH)	1759	https://www.ncbi.nlm.nih.gov/mesh/
	Total		27 056 367	

**Organisms:** Organisms in SPOKE are identified by their NCBI Taxonomy ID (Schoch *et al.*, 2020). Species of interest are determined by several different sources: bacterial information from KEGG (Kanehisa and Goto, 2000) and MetaCyc (Caspi *et al.*, 2016) and pathogenic species from PathoPhenoDB (Kafkas *et al.*, 2019).

**Proteins:** The source for all protein information in SPOKE is UniProt (Pundir *et al.*, 2017). Both SwissProt (reviewed) and TrEMBL (unreviewed) proteins are retrieved for all of the leaf Organisms.

In addition to Protein-cleavesto-Protein edges, we also incorporate data from several different sources to create Protein-interacts-Protein edges. For human proteins, the primary source for this information is STRING (Szklarczyk *et al.*, 2019). In addition, all IntAct (Orchard *et al.*, 2014) protein–protein interactions are retrieved for all proteins in SPOKE.

Finally, Protein nodes are linked to the Organism node (representing the species for that Protein) by creating Organism-encodes-Protein edges. These edges are created from the NCBI Taxonomy ID that is associated with the protein information loaded from UniProt.

**Genes:** Human gene information is imported from NCBI Gene (Maglott *et al.*, 2011). For human genes, the gene is linked to the encoded protein using Gene-encodes-Protein edges by using the UniProt gene information described above.

**Diseases:** SPOKE uses the Human Disease Ontology (Schriml *et al.*, 2012) as the primary identifier for Disease. The disease ontology information is read from the latest OBO file, downloaded weekly from https://github.com/DiseaseOntology/HumanDiseaseOntology and, in addition to creating the Disease nodes, we also create the standard ontology links Disease-isa-Disease. The DISEASES database (Pletscher-Frankild *et al.*, 2015) is downloaded and parsed to provide Disease-associates-Gene edges, which include the sources, scores and confidence values from the DISEASES database as edge attributes. In addition to information from the DISEASES database, both OMIM (Amberger *et al.*, 2015, 2019) and the GWAS Catalog (Buniello *et al.*, 2019) are used to provide Disease-associates-Gene edges. Furthermore, the GWAS Catalog uses the Experimental Factor Ontology (Malone *et al.*, 2010) to encode disease information. The GWAS lead variant *P*-value is added to the edge as a property.

In addition to Disease-associates-Gene edges, two more disease-related edges are included in the core: Organisms-causes-Disease and Disease-resembles-Disease. To create Organisms-causes-Disease edges, data from PathoPhenoDB (Kafkas *et al.*, 2019) are imported, which links human pathogens to the associated disease. Disease-resembles-Disease edges are based on the co-occurrence of disease terms (based on MeSH) in PubMed. Co-occurrence is scored based on Fisher’s exact test to provide both odds ratios and *P*-values, which are stored as edge properties along with the number papers that have both terms and the enrichment (measured as the number of papers with both terms over expected number based on a random distribution).

**Compounds:** For compound information, we chose to import ChEMBL (Mendez *et al.*, 2019). In addition, DrugBank (Wishart *et al.*, 2018) is used to include compounds that might not be present in ChEMBL. We also add Compound-binds-Protein edges from ChEMBL as well as BindingDB (Chen *et al.*, 2001).

ChEMBL and DrugCentral (Avram *et al.*, 2021; Ursu *et al.*, 2017) both provide information about the disease targets of drugs. Disease information is stored by ChEMBL using the MeSH identifier.

Finally, we import data from the Connectivity Map project (Subramanian *et al.*, 2017) which provides information linking perturbagens, including compounds and genes, to the regulatory effects on genes. In order to create the edges, we process the L1000 data to derive consensus signatures following the method outlined in Himmelstein *et al.* (2017).

As we continue to evaluate various databases that contain biological or biomedical data of interest, we integrate databases into SPOKE that augment the core with useful information but do not significantly introduce entire new ways of looking at SPOKE. Five examples of this include adding Gene Ontology (Ashburner *et al.*, 2000) annotations for CellularComponent, MolecularFunction and BiologicalProcess; ProteinDomain and ProteinFamily from PFAM (Finn *et al.*, 2014); the Uberon ontology (Mungall *et al.*, 2012) for Anatomy and CellTypes from the Human Protein Atlas (Thul and Lindskog, 2018); PharmacologicClass of Compounds from DrugCentral (Avram *et al.*, 2021); and Symptom from MeSH terms.

The InterPro database (Blum *et al.*, 2021) is used to provide ProteinDomain-partof-Protein edges, which provides the linkage between ProteinDomain and the SPOKE core. The ProtCID database (Xu and Dunbrack, 2020) provides information about known interactions between protein domains as well as between protein domains and compounds.

Finally, the Bgee (Bastian *et al.*, 2021) resource is used to determine differential expression of genes across tissues. This information is used to encode Anatomy-upregulates-Gene, and Anatomy-downregulates-Gene edges.

**Pathways:** Initially, we imported human pathway information from WikiPathways (Martens *et al.*, 2021) and Pathway Commons (Cerami *et al.*, 2011). These resources were used to add a Pathway node type, which is connected to Gene with Gene-participates-Pathway edges.

To import metabolic pathways, we read data from KEGG (Kanehisa and Goto, 2000), MetaCyc (Caspi *et al.*, 2016) and PATRIC (Wattam *et al.*, 2014). We use a reaction-centric model, adding a Reaction node that links to the metabolites with Reaction-consumes-Compound and Reaction-produces-Compound edges. A key part of the model is the addition of an EC node that links to the Reaction through an EC-catalyzes-Reaction edge. The EC node also links to the Proteins that have that EC using Protein-has-EC edges.

**Food:** The current version of SPOKE contains two food databases: FooDB (Scalbert *et al.*, 2011) and the Australian Food Composition Database from Food Standards Australia New Zealand. Two edges are derived from the databases, Food-contains-Compound and Food-contains-Nutrient. We are currently integrating the FoodOn (Dooley *et al.*, 2018), an ontology of foods that we will use to map foods from the various databases into a consistent ontology.

### 2.2 REST API

All of the nodes and edges discussed above are accessible through the SPOKE REST API. The API was designed primarily to support the Neighborhood Explorer graphical user interface (Fig. 3) but also provides reasonable access to the SPOKE database for other potential uses. The API can be roughly divided into three different parts: calls that return meta-information, calls that return information about nodes and calls that return networks. All API calls begin with the prefix: https://spoke.rbvi.ucsf.edu/api/v1/. The API is documented more fully at https://spoke.rbvi.ucsf.edu/swagger/. The metagraph call returns a cytoscape.js (Franz *et al.*, 2016) formatted JSON file that reflects the current SPOKE metagraph. The SPOKE call for getting information about nodes is the full-text search call search. The search call takes two arguments: a node type and a query term. This call uses the Neo4j full-text capability to quickly return a set of matching nodes of the indicated type that match that query, where the query is a lucene-formatted (Białecki *et al.*, 2012) query.

The SPOKE network calls are more complicated to allow more complicated filters and cutoffs. The three network calls all return cytoscape.js JSON networks. The sea call takes a SMILES (Weininger, 1988) string or a ZINC (Irwin and Shoichet, 2005) identifier as an argument and returns the SEA (Keiser *et al.*, 2007) network. The neighborhood call is similar to the node call and takes node_type, attribute and value arguments. See https://spoke.rbvi.ucsf.edu/swagger/ for more information. The final network call is the expand call, which takes as its input a node type and an internal node ID to expand along with a list of existing node identifiers.

## 3 Results

SPOKE is a knowledge graph connecting information from 41 biomedical databases. The current release contains more than 27 056 367 nodes of 21 different types (Table 1) and 53 264 489 edges of 55 types (Supplementary Table S1). SPOKE uses 11 different ontologies as a framework to organize and connect data in a semantically meaningful manner.

SPOKE strategically collects content from a range of biomedical data sources (i.e. providers of facts or established knowledge). In order to enhance its relevance to human health, SPOKE focuses on experimentally determined information. Thus, computational predictions and text mining from the literature are not currently prioritized. SPOKE is implemented as a Neo4j Community instance and built weekly from scratch by a series of custom python scripts which download each resource, check for integrity and completeness, and then create a ‘root table’ of nodes and edges. Finally, a Cypher script is used to upload the root table into Neo4j (Supplementary Fig. S1). Graph queries are translated by a REST API and users can submit searches directly via the API or via the graphical user interface (Neighborhood Explorer).

**Fig. 2.**
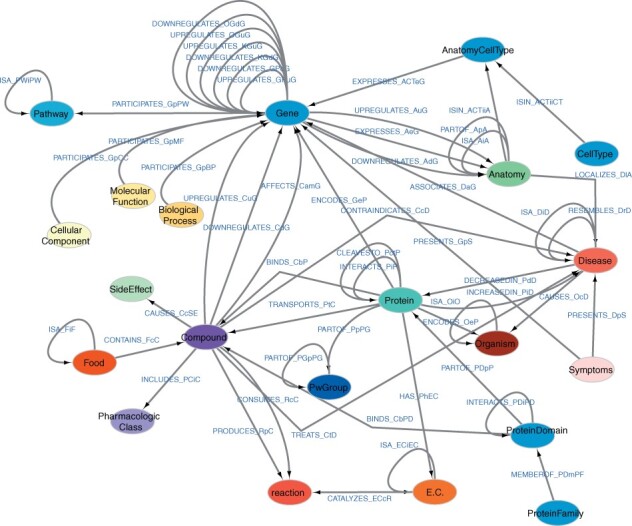
SPOKE Metagraph. Nodes denote biomedical concepts and links show how data is related and connected in the graph. For details on the node and edge nomenclature, please refer to Table 1 and Supplementary Table S1

The SPOKE metagraph (Fig. 2) shows all node types connected by biologically meaningful, semantic relationships. Both nodes and edges retain source properties that are exposed to the user and include provenance, context, descriptions, etc. If available, additional details are encoded as edge properties, such as association *P*-value and odds ratio (or Beta value) for an associated genetic variant, etc.

### 3.1 Ontologies

Ontologies are used to provide hierarchical structure to the graph, which enables anchoring of additional concepts and facilitates logical navigation. SPOKE also uses ontologies to mark up the datasets coming into the knowledge graph so that the data can be linked consistently across all other datasets. Whenever practical, SPOKE also adheres to the Biolink model. While not strictly an ontology, the Biolink model aims at standardizing the types and relational structures present in biomedical knowledge graphs.

### 3.2 Identifiers

For each type of node in SPOKE, a unique identifier must be chosen. While several different identifiers can be found for the same concept, one identification is selected as primary (SPOKE uses Ensembl). To enable cross-referencing, additional identifiers available for a given concept are kept as node properties.

### 3.3 Modeling

To preserve and make optimal use of available information, SPOKE considers genes and proteins as separate concepts (genes and transcripts remain unified). This distinction is particularly useful to describe protein isoforms, to properly map disease associations to genes, to accurately describe gene–gene regulations, and to distinguish drug–protein interactions from drug–gene (transcript) regulation. In most cases, data are downloaded and integrated ‘as is’, thus no modification to the source data is introduced.

### 3.4 Database download and update scripts

SPOKE is supported by a collection of Python scripts that identify the URL for the resource, downloads data tables, matches identifiers and creates nodes and edges between corresponding concepts.

### 3.5 Graphical user interface: the SPOKE Neighborhood Explorer

**Fig. 3.**
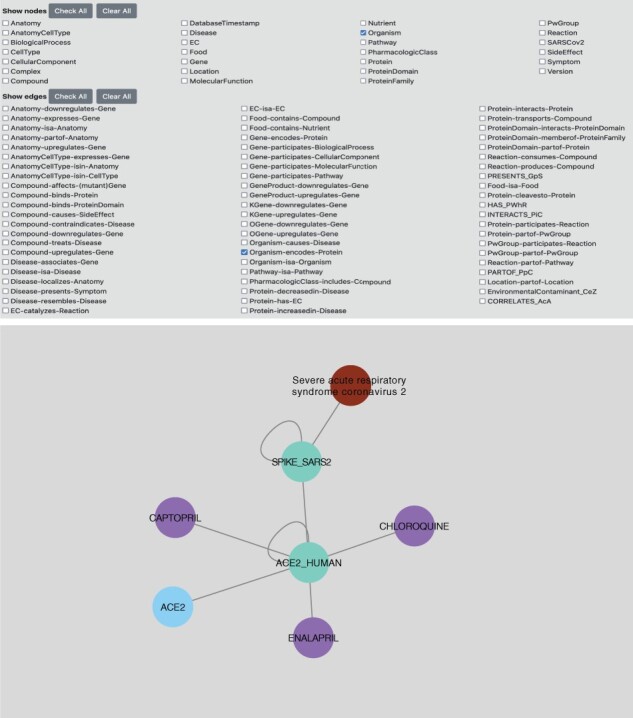
A view of the SPOKE Neighborhood explorer. The top panel shows the controls that allow a user to select nodes/edges for expansion as well as other key parameters. The bottom panel shows an example of the graph neighbors of the SARS-CoV-2 Spike protein (light blue), which includes three human genes (blue) and the proteins they encode (green). One such protein (ACE2_HUMAN) acts as the virus receptor in humans and has edges connecting it to three compounds (two of them FDA-approved and one -ORE-100- in experimental phase)

SPOKE can be accessed via the Neighborhood Explorer (Fig. 3). The SPOKE Neighborhood Explorer is a simple web interface (https://SPOKE.rbvi.ucsf.edu) that allows a researcher to query a given drug, disease, gene or protein and returns its neighbors in graph space—with precise controls (i.e. options) over the kind of nodes and edges that will be retrieved to the user, and a mouse-over function that displays the node/edge metadata (including its provenance). To preserve integrity of the original databases and prevent redistribution of content, SPOKE is not available as a bulk download.

### 3.6 Uses for SPOKE

**Fig. 4.**
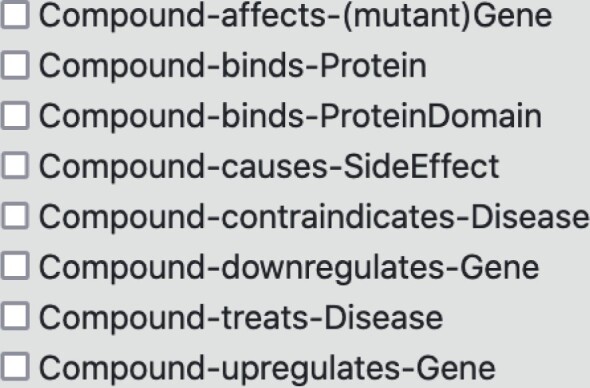
Available relationships for compounds. Example of user options to select different relationships for compounds. Compound binds protein (BINDS_CbP) reflects molecular interactions obtained from ChEMBL and BINDINGdb. Compound causes side effect (CAUSES_CcSE) relationships are retrieved from SIDER. Compound contraindicates disease (CONTRAINDICATES_CcD) and Compound treats disease relationships are obtained from ChEMBL. Compound downregulates gene (DOWNREGULATES_CdG) and Compound upregulates gene (UPREGULATES_CuG) are obtained from LINCS1000

**Drug discovery capabilities**: Compounds with therapeutic evidence (FDA-approved) or under experimentation, can be directly searched via their ChEMBL identifier or by typing in free text. Relationships to diseases (ChEMBL and DrugCentral), protein binding (ChEMBL and bindingDB), side effects (SIDER) or gene regulation (LINCS L1000) are available for selection (Fig. 4). Predicted binding to human proteins [pre-computed by the SEA algorithm (Keiser *et al.*, 2007)] can be retrieved by entering the compound’s SMILES ID. Starting from a SPOKE search, advanced graph analytic and machine learning approaches can be employed to use multi-node drug neighborhoods as a ‘functional fingerprint’ to complement its molecular profile for drug discovery or repurposing approaches.

**Anatomy-driven searches:** The class hierarchy view among anatomical terms can be explored by expanding any term using the subsumption relationships (Anatomy-isa-Anatomy, UBERON).

The edges Anatomy-partof-Anatomy describe relationships between Anatomy nodes (also from Uberon) indicating physical inclusion, for example, ‘brain’ is a part of ‘central nervous system’.

**Fig. 5.**
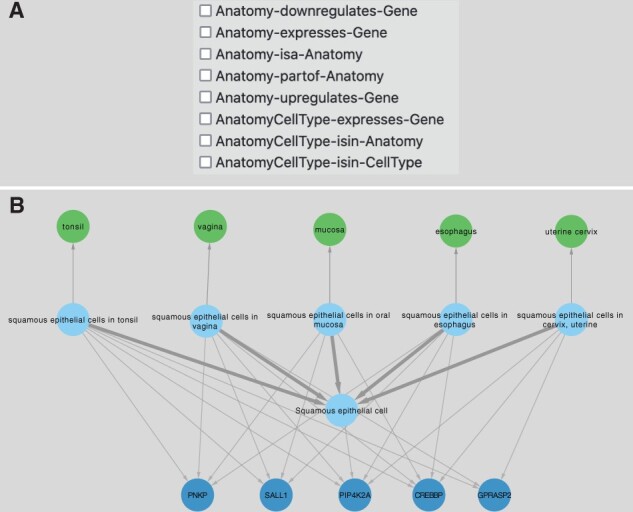
Anatomy-cellType-gene (protein) relationships. (**A**) An example of user options to select and control visualization of anatomy nodes in SPOKE. (**B**) An example of how the cell type squamous epithelial cell (blue—from cell ontology-), can be present in multiple organs/tissues (green—from UBERON). A hybrid node (AnatomyCellType) was created to represent the conditional statement that a given cell type can be found in multiple different tissues/anatomies. This allowed for the representation of immunostaining of histological specimens (from The Protein Atlas). For example, squamous epithelial cells in Tonsil express SALL1, but squamous epithelial cells in mucosa do not

Nominal or enriched gene expression information by each anatomy can be retrieved by Anatomy-expresses-gene or anatomy-upregulates-gene edges (Bgee). Cell types are connected to anatomies via intermediate AnatomyCellType nodes and AnatomyCellType-isin-Celltype edges. This modeling strategy was implemented to disambiguate cases in which the same cell type is found in different organs but they express different genes in each case [e.g. squamous epithelial cells can be found in several anatomies and their expressed genes/protein profiles can be different (Fig. 5)].

**Fig. 6.**
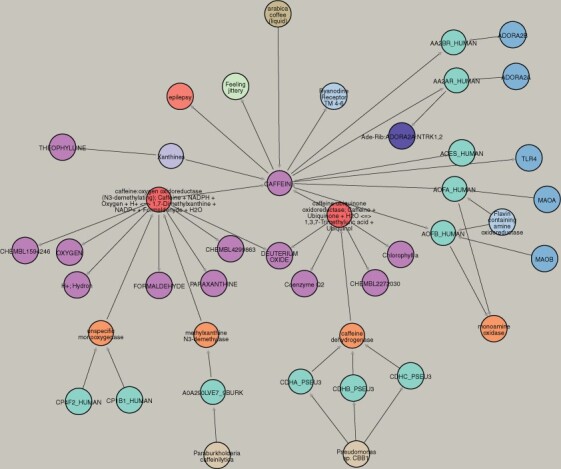
A search for Coffee reveals molecular, pharmacological and metabolic pathways of caffeine. A multi-step search for coffee can provide a deep understanding of its relationship to human metabolism. In this example, Arabica coffee (food) contains caffeine (compound), which, together with theophylline, is a xanthine (pharmacological class). In addition, caffeine binds two adenosine receptors (AA2AR and AA2BR), encoded by the genes ADORA2A and ADORA2B, acetylcholinesterase (ACES) and monoamine oxydase A (ACFA) and B (ACFB). Caffeine also binds the protein domain Ryanodine receptor, upregulates the gene TLR4, causes a feeling jittery side effect and is contraindicated in epilepsy. In the left-hand side of the figure, two metabolic reactions that consume caffeine are depicted. A mono-oxygenase catalytic activity is denoted for cytochrome P450 complex in humans, and a methylxanthine demethylase activity in *Pseudomonas putida*. A dehydrogenase activity is carried out by enzymes in *Pseudomonas* sp. CBB1

**Food-driven searches:** With the incorporation of FooDB and Australian Food Composition Database, thousands of edges connect chemicals to common foods. When available, a numeric quantity describes the amount as an edge property. This is useful when connecting foods with metabolic reactions or components of the gut microbiota. Indeed, a SPOKE search can be initiated with any available foods and use a combination of Extend and Options to display a complete picture of the role of its neighborhood. For example, a user can start a search with the term ‘(arabica) coffee’ and bring the compound caffeine as one of its components (Fig. 6). An unrestricted extension of caffeine brings nodes of different types, including proteins (Adenosine receptors, acetylcholinesterase and monoamine oxidases) known to bind this compound. As some of them are enzymes (MAO-A and MAO-B), a connection to the corresponding E.C. (Monoamineoxidase) can be retrieved. In addition, protein domains (light blue) from each protein can be retrieved. Caffeine is also connected to the gene TLR4 (by an edge Compound_upregulates_gene), as reported by LINCS L1000. Additional information is available for caffeine, such as its pharmacological class (xantines), associated side effects (e.g. feeling jittery) and disease contraindications (e.g. epilepsy). In addition, caffeine is linked to a series of metabolic reactions (red nodes), some of which are endogenous (monooxygenase and Cytochrome P450) and some are bacterial (e.g. a demethylase and a dehydrogenase) corresponding to *Pseudomonas putida* (Yu *et al.*, 2009). Thus, SPOKE was able to reconstruct a large body of knowledge by linking information deposited in multiple databases (Fig. 6).

**Fig. 7.**
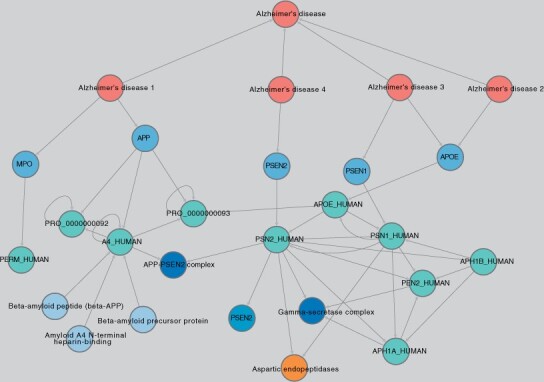
Main types of Alzheimer’s disease and their relationships to symptoms, genes, proteins, domains and families. Four subtypes of Alzheimer disease are depicted, each with its corresponding genetic association. Type 1 is related to variation/mutation in MPO and APP, Type 2 is related to APOE, Type 3 is related to APOE and PSEN1, and Type 4 is related to PSEN2 (blue). The corresponding proteins encoded by those genes are also depicted (teal). The enzymatic proteolysis of APP into the different amyloid peptides by the secretase complex (encoded by presenilins 1 and 2) is depicted at the bottom of the figure

**Disease-driven searches:** Diseases can be explored by entering a DOID or text and selecting any of the available Options, which include relationships to genes, symptoms, indications, similarity and anatomy (in addition to exploring the disease ontology). For example, it is possible to search for Alzheimer’s disease (AD, DOID: 10652), and retrieve just its symptoms (PubMed) and all sub-types of the disease described in the DO (AD1, AD2, etc.) (Fig. 7). An extension to this search can be performed to bring genes associated with each disease subtype (GWAS Catalog, OMIM and DISEASES), the proteins these genes encode (NCBI Gene) and their domains and families (PFAM). Entire classes of diseases can be explored at once, by leveraging the disease ontology. For example, all Mendelian or metabolic diseases can be retrieved in a single query. For a given metabolic disease, it is possible to explore relationships to gene, protein, enzymatic activity, all the way down to the metabolic reaction affected by the gene defect. This strategy is particularly useful when searching for compounds that can reverse the damage either by reducing degradation or by increasing production of the affected metabolite.

## 4 Discussion

Knowledge can be considered an emergent property of the interconnected web of trusted information and known facts. The space of the ‘unknown knowns’ is growing fast and remains vastly underexplored. Concretely, in order to effectively mine them, we must ‘connect the dots’ from several information sources. We argue that when heterogeneous networks are connected at a massive scale, new knowledge can be extracted as an emergent property of the network. In this article, we present SPOKE, a large biomedical knowledge graph that amalgamates data and information from a large spectrum of databases ranging from molecular to physiological processes.

SPOKE has been used for a variety of biomedical applications including drug repurposing (Himmelstein and Baranzini, 2015), disease prediction and interpretation of transcriptomic data (Himmelstein and Baranzini, 2015), among others. More recently, we developed an algorithm to embed electronic health records onto SPOKE, which, when combined with machine learning techniques, enables a wide range of applications relevant to precision medicine (Nelson *et al.*, 2019, 2022). This approach uses an original embedding method based on the Page rank algorithm that enables the creation of concept-specific vectors (PSEV) trained in millions of de-identified electronic health records. These vectors describe cohorts of patients that share one specific concept (e.g. patients treated with the drug Metformin or patients with tremor as a symptom). Each of these embeddings represents the importance of each node in SPOKE for that cohort, based on the training data, and can later be combined to represent the status of a given patient at a particular point in time. For details, see Nelson *et al.* (2019). This approach has been successfully implemented to predict a diagnosis of multiple sclerosis with up to 83% accuracy 3 years before the first disease code was found in the EHR (Nelson *et al.*, 2022). A similar approach is now being used to predict diagnosis of other chronic diseases, such as Parkinson’s and Alzheimer.

A number of biomedical knowledge graphs exist, but without clear standards for their representation and modeling, a wide variety of strategies have been implemented. Naturally, such knowledge graphs have been difficult to create, as they require deep expertise in a variety of domains. In particular, biomedicine has been slow to adopt this potentially transformative approach, in part due to the complexity of the underlying information. While some focus on experimentally determined information, others include primary data, literature mining and predicted relationships. In addition, these resources can be implemented as property graphs or using RDF (triples) representation (DataCommons https://www.datacommons.org/), which largely determines the range of applications they can be used for. Finally, some biomedical graphs are built using semi-automated methods (Rossanez *et al.*, 2020; Santos *et al.*, 2022), and others like SPOKE, Robokop (Fecho *et al.*, 2021) and the comparative toxicogenomics database (Mattingly *et al.*, 2006), CTD, require extensive manual curation (Table 2 illustrates key features of some of the most relevant biomedical graphs available).

**Table 2.** Comparison of biomedical knowledge graphs

	SPOKE	CGK	ROBOKOP	ARAX	KeyGEN	CTD
User friendly	+++	+	++	+	+	+++
Experimental info rich	+++	+	+	+	n/a	+++
Literature rich	+	+++	++	+++	ONLY	++
Ontologies	+++	+	++	++	++	+
Food info	YES	YES	NO	YES	n/a	NO
Metabolic info	YES		NO	NO	n/a	NO
Microbiome info	YES		NO	NO	n/a	NO
Full analytics workbench	NO	YES	NO	YES	NO	NO
Automatically generated	NO	NO	NO	NO	YES	NO
Installation needed	NO	YES	YES	NO	NO	NO

The Biomedical Data Translator project (Translator, for short) (https://ncats.nih.gov/translator) is a novel and ambitious undertaking by the National Institutes of Health’s National Center for Advancing Translational Sciences involving a large and collaborative cadre of scientists from a variety of scientific domains including semantic representation, computer science and biomedical experts. The Translator project aims at developing a comprehensive, relational, N-dimensional Biomedical Data Translator that integrates multiple types of existing data sources, including objective signs and symptoms of disease, drug effects and intervening types of biological data relevant to understanding pathophysiology. SPOKE is one of the knowledge providers of the Translator project.

The National Science Foundation’s Convergence Accelerator Program catapulted the development of SPOKE and other open knowledge graphs in the content of track A, which started in 2019. The program prompted a 10× growth in SPOKE in terms of number of nodes, edges and types of information incorporated. Current applications in development include graph traversal, embeddings and drug repurposing efforts, among others.

Machine and deep learning models such as neural networks have traditionally been considered ‘black boxes’, capable of delivering predictions, but in and of themselves, no new knowledge. This perceived limitation has slowed down their adoption in a range of chemical and biological contexts, under the sensible argument that a technique a scientist, clinician or engineer cannot understand will in turn provide no guarantee of correctness in a true discovery context. Similarly, biomedicine, and human health in general, has been a ‘black box’ field for predictions and prognoses. In this context, SPOKE can be used to predict biomedical outcomes in a biologically meaningful manner thus representing ‘clear box’ (i.e. explainable) models. With SPOKE, the paradigm of knowledge graphs—amply proven in Search—is ready to be tested and ultimately applied in biomedicine.

## Supplementary Material

btad080_Supplementary_Data

## Data Availability

To preserve integrity of the original databases and prevent redistribution of content under multiple licenses, SPOKE is not available as a bulk download.
